# Copy number variation at leptin receptor gene locus associated with metabolic traits and the risk of type 2 diabetes mellitus

**DOI:** 10.1186/1471-2164-11-426

**Published:** 2010-07-12

**Authors:** Jae-Pil Jeon, Sung-Mi Shim, Hye-Young Nam, Gil-Mi Ryu, Eun-Jung Hong, Hyung-Lae Kim, Bok-Ghee Han

**Affiliations:** 1 Division of Biobank for Health Sciences, Korea National Institute of Health, Korea Centers for Disease Control and Prevention, 194 Tongil Ro, Seoul, 122-701, Korea; 2 Center for Genome Science, Korea National Institute of Health, Korea Centers for Disease Control and Prevention, 194 Tongil Ro, Seoul, 122-701, Korea

## Abstract

**Background:**

Recent efforts have been made to link complex human traits and disease susceptibility to DNA copy numbers. The leptin receptor (LEPR) has been implicated in obesity and diabetes. Mutations and genetic variations of *LEPR *gene have been discovered in rodents and humans. However, the association of DNA copy number variations at the *LEPR *gene locus with human complex diseases has not been reported. In an attempt to study DNA copy number variations associated with metabolic traits and type 2 diabetes mellitus (T2DM), we targeted the *LEPR *gene locus in DNA copy number analyses.

**Results:**

We identified DNA copy number variations at the *LEPR *gene locus among a Korean population using genome-wide SNP chip data, and then quantified copy numbers of the E2 DNA sequence in the first two exons overlapped between *LEPR *and *LEPROT *genes by the quantitative multiplex PCR of short fluorescent fragment (QMPSF) method. Among the non-diabetic subjects (n = 1,067), lower E2 DNA copy numbers were associated with higher fasting glucose levels in men (*p *= 1.24 × 10^-7^) and women (*p *= 9.45 × 10^-5^), as well as higher total cholesterol levels in men (*p *= 9.96 × 10^-7^). In addition, the significant association between lower E2 DNA copy numbers and lower level of postprandial 2hr insulin was evident only in non-diabetic women, whereas some obesity-related phenotypes and total cholesterol level exhibited significant associations only in non-diabetic men. Logistic regression analysis indicated that lower E2 DNA copy numbers were associated with T2DM (odds ratio, 1.92; 95% CI, 1.26~2.96; p < 0.003) in our nested case-control study. Interestingly, the E2 DNA copy number exhibited a negative correlation with LEPR gene expression, but a positive correlation with LEPROT gene expression.

**Conclusions:**

This work suggests that a structural variation at the *LEPR *gene locus is functionally associated with complex metabolic traits and the risk of T2DM.

## Background

Leptin and the leptin receptor (LEPR) are involved in satiety and energy expenditure via central and peripheral mechanisms. The primary site of leptin action is the hypothalamus where the leptin receptor interacts with the adipocyte-derived leptin signal to regulate appetite, energy balance, and metabolism. LEPRs also regulate energy homeostasis in peripheral tissues including skeletal muscle, liver, pancreas, and adipose tissue. Leptin prevents obesity via LEPRs by stimulating glucose uptake and fatty acid oxidation in skeletal muscle and liver [1-3], and inhibits insulin secretion of pancreatic β-cells [4].

Mutations and genetic variations of the *LEPR *gene have been discovered in rodents and humans. Sequencing-based detection of *LEPR *mutations demonstrated that frame-shift or missense mutations of the *LEPR *gene caused obesity, pituitary dysfunction, hyperphagia, or hypogonadism in humans [5,6]. In rodents, leptin receptor gene mutations resulted in obesity, hyperglycemia, hyperinsulinemia, and reduced fertility [7,8]. Common genetic variants (e.g., SNPs) at the *LEPR *gene locus have been associated with obesity, hyperinsulinemia, type 2 diabetes mellitus (T2DM), and variations in leptin levels in different populations. For example, three non-synonymous SNPs (Arg109Lys, Arg223Gln, and Lys656Asn) have been evaluated for association studies [9-13]. Therefore, most association studies of *LEPR *polymorphisms have been performed on SNPs or deletion/insertion polymorphisms (DIPs). However, the association of DNA copy number variations (CNVs) at the *LEPR *gene locus with human diseases has not yet been reported.

In recent years, comprehensive human CNV maps were assembled using various experimental platforms [14-16]. While the growing number of genome-wide association data sets can be utilized to detect clinically-relevant CNVs, absolute copy number information cannot be easily determined by current quantitative assays, with the exception of Fiber-FISH. Nonetheless, recent studies have shown that CNVs are implicated in human diseases including glomerulonephritis (FCGR3B) [17], HIV-1/AIDS (CCL3L1) [18], bipolar disorder and schizophrenia (GLUR7, CACNG2 and AKAP5) [19], neoplasia (14q12) [20], psoriasis (DEFB) [21], and myocardial infarction (C4B) [22].

## Results

**Figure 1:**
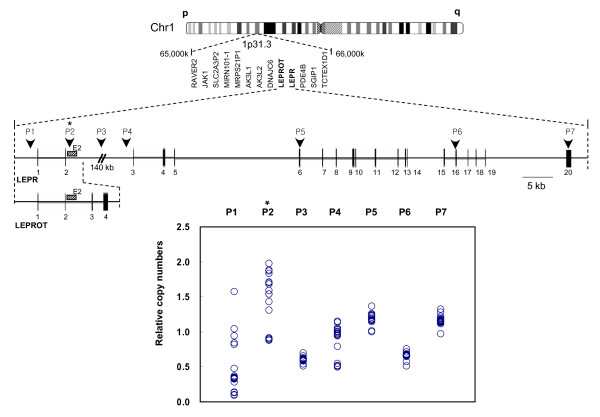
**Schematic diagram of CNVs at the LEPR gene locus**. Genomic structures of *LEPR *and *LEPROT *genes, including exons, are shown. E2 DNA content relative to the Factor VIII gene from 20 subjects is displayed in the lower panel. Primer target sites (P1 to P7) for QMPSF are indicated along the *LEPR *sequence. P2* indicates the primer target site for detection of the initial copy number at the *LEPR *locus. P1 and P2 primer target sites were highly variable for copy number among the 19 individuals tested, but the other primer sites were not.

On employing a candidate gene approach to the association study of CNVs with diabetes and metabolic traits, we targeted the genomic locus of the LEPR gene encompassing approximately 200 kb of chromosome 1. DNA copy number analysis of the Affymetrix 50 K SNP array data showed that the Korean population exhibited CNVs at the LEPR gene locus (Additional file 1). Our QMPSF experiments generated continuous variables of DNA copy number at the *LEPR *locus for individuals (Additional file 2). Next, the experimental copy number information was transformed to dichotomized DNA copy numbers (low or high) for CNV association studies. An appropriate copy number value (low or high) was then assigned to each individual using either male or female median copy number value among non-diabetic subjects (n = 1,067) as the cutoff. The same cutoff value was used to dichotomize copy numbers among diabetes subjects in the nested case-control study. DNA copy numbers were determined using only a short PCR fragment (herein referred to the "E2 DNA", located near exon 2 of the LEPR gene, see Figure 1) of the entire LEPR gene sequence. To address the question as to whether this short sequence was representative of the whole LEPR gene, we investigated the CNV boundaries using PCR-based (QMPSF) and array-based (Affymetrix SNP array 5.0) analyses. Relative DNA copy numbers were obtained by QMPSF using primers targeting several regions along the LEPR gene sequence. QMPSF results showed that the copy number of the E2 DNA sequence was correlated to that of the promoter-containing exon region at the *LEPR *locus, but not to the other downstream exonic regions (Figure 1). On the other hand, CNV analysis of the Affymetrix SNP array 5.0 data revealed that the whole LEPR gene locus region was highly CNV-affected, with a varied length of CNV boundaries over the LEPR gene sequence (Additional file 1). This result indicated that the E2 DNA copy number did not represent DNA copy numbers of the whole LEPR gene, but a partial DNA sequence containing the LEPROT (leptin receptor overlapping transcript) gene at the *LEPR *locus.

**Figure 2:**
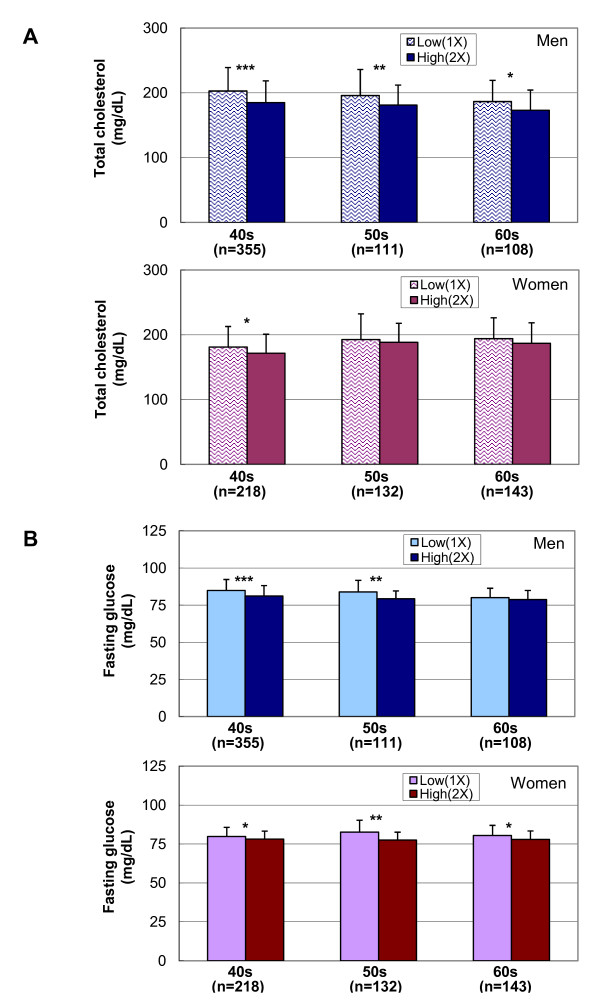
**Total cholesterol and fasting glucose levels for individuals with low or high copy numbers of the E2 DNA sequence**. Non-T2DM subjects were divided into different age groups (40 s, 50 s, and 60s) and their metabolic phenotypes were compared. (A) Total cholesterol levels. ***: *P *= 2.13 × 10^-6^, **: *P *= 4.33 × 10^-3^, *: *P *< 0.05. (B) Fasting glucose levels. ***: *P *= 1.72 × 10^-6^, **: *P *= 8.39 × 10^-4^, *: *P *< 0.03.

Next, we tested the statistical significance of the association of the E2 DNA copy number with metabolic traits. In male non-diabetic subjects, significant differences for some demographic characteristics were observed between low (1X) and high (2X) copy number individuals (Table 1). Fasting glucose and total cholesterol levels were higher in individuals with low copy numbers of the E2 DNA at LEPR gene locus in three different age groups of male individuals (Figure 2). The association of low copy numbers with higher levels of obesity-related phenotypes and total cholesterol levels remained significant in linear regression analysis with age-and BMI-adjustments followed by Bonferroni correction for multiple testing among male individuals (Table 1). Among female subjects, E2 DNA copy numbers were also associated with levels of fasting glucose and postprandial 2hr insulin. On the other hand, beneficial phenotypes for obesity-related traits and total cholesterol levels were associated with higher DNA copy numbers (2X) of the *LEPR *gene locus in male subjects.

**Table 1:** Association of LEPR CNVs with metabolic traits in nondiabetic subjects.

Variables	Men (n = 574)					Women (n = 493)				
	
	Low CN (1X) (n = 287)	High CN (2X) (n = 287)	*P *value*	*P *value** (adjusted)	*P *value*** (corrected)	Low CN (1X) (n = 246)	High CN (2X) (n = 247)	*P *value*	*P *value** (adjusted)	*P *value*** (corrected)
Age (year)	49.1 ± 8.4	50.7 ± 8.6	0.026	-	-	52.9 ± 9.0	52.5 ± 8.6	0.505	-	-
Height (cm)	167.2 ± 5.7	167.6 ± 6.1	0.479	0.074	ns	153.9 ± 5.9	153.7 ± 5.6	0.887	0.716	ns
Weight (kg)	67.1 ± 9.4	66.6 ± 9.4	0.431	0.082	ns	57.3 ± 8.6	57.6 ± 7.7	0.814	0.690	ns
Pulse (counts)	61.0 ± 6.5	61.4 ± 7.2	0.418	0.439	ns	64.2 ± 7.1	63.8 ± 6.8	0.656	0.719	ns
Systolic blood pressure (mmHg)	114.0 ± 14.7	114.6 ± 13.9	0.575	0.776	ns	115.5 ± 17.6	115.8 ± 15.7	0.800	0.608	ns
Diastolic blood pressure (mmHg)	75.1 ± 11.2	74.5 ± 10.6	0.512	0.502	ns	72.7 ± 11.1	73.4 ± 10.0	0.401	0.286	ns
Distal radius Z	0.16 ± 1.23	0.48 ± 1.35	2.85E-03	0.013	ns	0.91 ± 1.48	1.08 ± 1.53	0.305	0.295	ns
Midshaft tibia Z	0.47 ± 1.06	0.68 ± 1.18	0.025	0.071	ns	-0.36 ± 1.30	-0.39 ± 1.31	0.768	0.834	ns
Waist circumference (cm)	82.2 ± 7.3	81.4 ± 7.1	0.194	0.353	ns	81.0 ± 9.3	81.7 ± 9.2	0.531	0.311	ns
Hip circumferences (cm)	93.9 ± 5.6	92.2 ± 6.2	3.67E-04	6.07E-04	**1.76E-02**	91.9 ± 6.3	91.7 ± 6.0	0.849	0.455	ns
Waist-to-hip ratio (WHR)	0.88 ± 0.06	0.88 ± 0.06	0.080	0.074	ns	0.88 ± 0.08	0.89 ± 0.08	0.380	0.182	ns
Body fat (%)	20.5 ± 4.9	19.4 ± 4.5	6.01E-03	1.03E-03	**2.99E-02**	29.6 ± 5.8	30.3 ± 4.8	0.201	0.072	ns
Visceral fat (%)	0.89 ± 0.04	0.88 ± 0.04	9.45E-03	1.02E-06	**2.95E-05**	0.89 ± 0.05	0.89 ± 0.05	0.898	0.814	ns
Obesity degree (%)	112.2 ± 12.7	110.2 ± 12.5	0.051	5.85E-04	**1.70E-02**	119.9 ± 17.2	120.8 ± 15.1	0.686	0.643	ns
Body mass index (BMI) (kg/m^2^)	23.9 ± 2.7	23.6 ± 2.7	0.158	-	-	24.2 ± 3.3	24.4 ± 2.9	0.715	-	-
Total cholesterol (mg/dL)	199.1 ± 36.7	181.7 ± 32.8	3.23E-09	3.43E-08	**9.96E-07**	188.0 ± 34.3	180.9 ± 31.0	2.25E-02	2.50E-02	ns
HDL-cholesterol (mg/dL)	45.6 ± 8.5	44.4 ± 9.5	0.119	0.056	ns	47.7 ± 9.9	45.1 ± 9.5	7.40E-03	5.51E-03	ns
Triglyceride (mg/dL)	146.9 ± 86.1	151.4 ± 82.5	0.521	0.323	ns	122.0 ± 68.7	137.1 ± 73.2	0.016	9.11E-03	ns
C-reactive protein (mg/dL)	0.19 ± 0.31	0.20 ± 0.21	0.601	0.593	ns	0.15 ± 0.24	0.21 ± 0.52	0.232	0.220	ns
WBC (10^3^/μL)	6.6 ± 1.7	6.4 ± 1.7	0.333	0.499	ns	5.8 ± 1.5	5.6 ± 1.4	0.099	0.083	ns
RBC (10^6^/μL)	4.8 ± 0.4	4.7 ± 0.4	3.03E-03	0.029	ns	4.1 ± 0.3	4.1 ± 0.3	0.350	0.322	ns
Hemoglobin (g/dL)	14.7 ± 1.0	14.6 ± 1.1	0.191	0.552	ns	12.5 ± 1.3	12.4 ± 1.1	0.677	0.686	ns
Hemocritat	44.4 ± 3.2	44.1 ± 3.4	0.158	0.456	ns	37.9 ± 3.4	37.7 ± 3.5	0.348	0.357	ns
HbA1C (%)	5.5 ± 0.3	5.5 ± 0.3	0.853	0.584	ns	5.5 ± 0.3	5.4 ± 0.3	0.409	0.495	ns
Insulin_0hr (μIU/ml)	6.5 ± 4.7	6.2 ± 2.6	0.283	0.474	ns	7.6 ± 5.1	7.8 ± 3.5	0.644	0.066	ns
Insulin_1hr (μIU/ml)	32.0 ± 27.2	34.5 ± 33.2	0.324	0.195	ns	29.5 ± 26.9	37.2 ± 34.1	6.66E-03	5.38E-03	ns
Insulin_2hr (μIU/ml)	18.9 ± 18.2	18.9 ± 19.1	0.994	0.713	ns	22.1 ± 19.8	29.1 ± 24.0	8.55E-04	7.93E-04	**2.30E-02**
Glucose_0hr (mg/dL)	84.0 ± 7.5	80.3 ± 6.5	3.34E-10	4.26E-09	**1.24E-07**	80.7 ± 6.7	77.9 ± 5.2	3.37E-06	3.26E-06	**9.45E-05**
Glucose_1hr (mg/dL)	127.4 ± 34.9	128.3 ± 36.8	0.741	0.983	ns	121.7 ± 34.9	121.1 ± 31.4	0.675	0.754	ns
Glucose_2hr (mg/dL)	94.9 ± 21.4	91.1 ± 20.6	0.030	0.037	ns	103.0 ± 18.9	101.3 ± 18.9	0.200	0.200	ns
HOMA-IR	1.41 ± 1.09	1.39 ± 0.59	0.835	0.928	ns	1.70 ± 1.15	1.8 ± 0.8	0.261	0.263	ns

*: P value: t-test

**: P value: age-, BMI-adjusted linear regression test (CN values)

***: P value: obtained by Bonferroni correction for multiple testing. ns indicates none-significance (cutoff p < 0.05).

The copy numbers of the E2 DNA sequence at the LEPR gene locus were used in the nested case-control study of T2DM, in which subjects (n = 137) were matched for age, sex, and BMI at a ratio of approximately 1:2 to control subjects (n = 258). Our case groups showed typical demographic characteristics to diabetes subjects (Additional file 3). For example, diabetic case subjects had higher triglyceride, higher total cholesterol, and lower HDL-cholesterol than those levels in non-diabetic subjects. Logistic regression analysis showed that low E2 DNA copy numbers were associated with T2DM (odds ratio, 1.92; 95% CI, 1.26~2.96), with a significance of *P *< 0.003 (Table 2).

**Table 2:** The nested case-control study of T2DM

Case-control	E2 DNA copy number groups			Odds ratio (OR)	CI (95%)	*P *value*
				
	Low CN (1X)	High CN (2X)	Sum			
T2DM case subjects (n = 135)	86 (63.7%)	49 (36.3%)	135 (100%)	1.92	1.26~2.96	0.003
non-T2DM control subjects (n = 258)	123 (47.7%)	135 (52.3%)	258 (100%)			

Total (n = 393)	209 (53.2%)	184 (46.8%)	393 (100%)			

*P *value*: Logistic regression analysis

**Figure 3:**
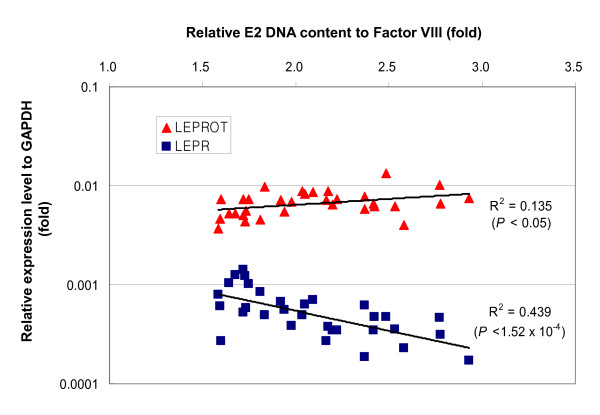
**Correlation of the E2 DNA copy number with *LEPR *and *LEPROT *transcription levels**. Expression levels of *LEPR *and *LEPROT *relative to *GAPDH *were compared to the relative E2 DNA content in 32 strains of LCLs. Relative E2 DNA content was calculated from QMPSF data in relation to DNA content at the Factor VIII gene locus in LCLs.

To study biological roles of the E2 DNA copy numbers, we compared mRNA expression levels of both LEPR and LEPROT genes with E2 DNA copy numbers in lymphoblastoid cell lines. The E2 DNA sequence exists immediately downstream of the overlapped exon 2 of the LEPR and LEPROT genes. Real-time PCR analysis showed a significant negative correlation of the E2 DNA copy number with the LEPR transcript level (R^2 ^= 0.439, *p *= 1.52 × 10^-4^), but a weak positive correlation with the LEPROT transcript level (R^2 ^= 0.135, *p *< 0.05) (Figure 3). Moreover, the *LEPR *expression level was inversely correlated with the LEPROT expression level (R^2 ^= 0.126, *p *< 0.05). This result suggests that higher copy numbers of the E2 DNA may contribute to higher transcriptional activity of the LEPROT gene by gene dosage effect, which may be accordingly responsible for downregulation of *LEPR *gene.

## Discussion

Our candidate gene approach to CNV association studies identified the E2 DNA copy number variation at the LEPR gene locus, which was associated with metabolic traits and the risk of T2DM. Lower copy numbers of the E2 DNA were associated with higher fasting glucose in both male and female non-diabetic subjects. However, obesity-related traits and total cholesterol levels exhibited significant association with the E2 DNA copy numbers only in male non-T2DM subjects whereas postprandial 2hr insulin level was significantly higher only in female non-T2DM subjects. These results suggest that the E2 DNA copy number variations at the *LEPR *gene locus may contribute to glucose homeostasis and energy metabolism in a different manner between male and female subjects.

In our CNV association study, we observed that the beneficial high E2 DNA copy number was associated with lower fasting glucose levels (hypoglycemia) in both genders which are beneficial in terms of diabetes. Similar results were reported for mouse models with LEPR signaling defects [23,24]. For example, hypothalamus and pancreas-specific LEPR signaling disruption led to fasting hypoglycemia, hyperinsulinemia, and hypertriglyceridemia, highlighting the important role of beta cells in LEPR mediated glucose homeostasis [24]. Hypothalamic LEPR KO^VMH ^mice fed a low fat diet also exhibited hypertriglyceridemia without concomitant hypercholesterolemia [25]. Neuronal LEPR deletion induces diabetic phenotypes such as body weight increase, adiposity, and hyperglycemia in proportion to hypothalamic LEPR deficiency [25]. In addition, antisense-RNA mediated adipocyte-specific reduction of LEPR leads to decrease in LEPR signaling, resulting in increased adipocyte, hypertriglyceridemia, and insulin resistance [26]. As presented in our association study, the high E2 copy number individuals shared similar phenotypes with LEPR deficient animal models. Moreover, human LEPR signaling mutations also caused obesity as well as impairments of pubertal development and growth hormone secretion [5]. Thus, it is conceivable that higher E2 DNA copy numbers may result in a decrease in LEPR signaling, which functions in a complex manner in different tissues.

Indeed, our expression analysis of LEPR gene in LCLs showed that the high E2 DNA copy number was significantly correlated with downregulation of the LEPR gene and concomitant upregulation of the LEPROT gene. Hypothalamic *LEPR *mRNA expression is indicative of functional LEP-LEPR signaling [27]. The *LEPR *and *LEPROT *transcripts share the first two exons, which are not translated in the LEPR gene [28]. Among the LEPR isoforms, the long form (LEPRb) is primarily expressed in specific nuclei of the hypothalamus, whereas the short isoform (LEPRa) is expressed in most tissues. These two isoforms of LEPR have identical extracellular domains but differ in the length of their intracellular domains, which contributes to their different capacity for signal transduction [29]. On the other hand, our CNV analyses of the Affymetrix SNP array 5.0 and QMPSF revealed a varied length of the CNV region at the *LEPR *gene locus. Also, we found that the E2 DNA copy numbers did not represent DNA copy numbers of the whole LEPR gene but only a partial sequence containing LEPROT gene. Thus, this finding suggests that E2 DNA copy numbers may negatively regulate LEPR gene expression through a gene dosage effect of *LEPROT*.

However, *LEPROT *overexpression decreases only cell surface expression of LEPR without modifying the total amount of LEPR [30]. In our study, the observed weak correlation of E2 DNA copy numbers with *LEPR *mRNA levels in LCLs might be ascribed to inappropriate selection of target tissues for *LEPR *and *LEPROT *expression studies. Thus, the correlation of high E2 DNA copy number with LEPROT gene expression may be more significant in an appropriate LEPR target tissues such as the hypothalamus, pancreatic β-cells, or adipocytes. Elucidating tissue-specific regulation of LEPR gene expression could explain the complex pattern of association of phenotypes with the E2 DNA copy number variation. It remains to be investigated whether the E2 copy number confers a gene dosage effect of LEPROT on LEPR signaling using an appropriate target tissue, and whether this occurs via either transcriptional regulation or intracellular transport of LEPR. Alternatively, the E2 DNA sequence may merely play a role as a direct negative transcriptional regulatory sequence for the LEPR gene expression.

## Conclusion

A prior CNV analysis of Affymetrix 50 K SNP and 5.0 array chip data indicated the potential presence of CNVs at the LEPR gene locus as well as a varied length of the CNV region. DNA copy number measurements at the LEPR gene locus and subsequent association studies revealed that lower copy numbers of the E2 DNA sequence around LEPROT gene were associated with detrimental phenotypes of metabolic traits (e.g., higher fasting glucose and total cholesterol levels) as well as the risk of T2DM. In addition, the E2 DNA copy number exhibited a negative correlation with LEPR gene expression but a positive correlation with LEPROT gene expression. More research may be warranted to determine if LEPROT expression impacts LEPR expression.

## Methods

### Study samples

Subjects described in this study include 1,204 individuals from the Korean Genome and Epidemiology Study (KoGES) for CNV detection and subsequent association studies. The KoGES is a community-based prospective cohort study of chronic diseases (e.g., diabetes and hypertension) and their relationship with potential risk factors or lifestyles, which was started in 2001. The study design, sampling, concept and consent have been previously described [31,32]. Briefly, a total of 10,038 individuals participated in baseline examinations. Approximately 86.6% (n = 8,693) and 67.8% (n = 6,085) of the baseline study subjects participated in the first and the second 2-year follow-up studies, respectively.

For the study of CNV association with metabolic traits, we randomly selected 1,067 non-diabetic subjects who did not develop T2DM during either baseline or two rounds of 2-year follow-up period from the KoGES. WHO criteria were used to define T2DM using an oral glucose tolerance test. Diabetes cases were defined as subjects who were determined to have T2DM at baseline, and then diabetes was diagnosed at least one time during two rounds of 2-year follow-ups. For the nested case-control study, T2DM cases (n = 137) were matched for sex, age±1, and BMI±0.1, with control subjects (n = 258) who did not have diabetes in either baseline or during the 2-year follow-ups. This study was approved by the institutional review boards of Korea Centers for Disease Control and Prevention.

### DNA copy number measurement

DNA samples used for DNA copy number measurement were provided by the National Biobank of Korea in Korea Centers for Disease Control and Prevention. We extracted CNV information for candidate genes (diabetes-and obesity-related) from the Affymetrix 50 K SNP chip data in order to estimate whether CNVs were detectable within or near these genes in the Korean population. The Affymetrix 50 K SNP chip data was obtained from the Korean HapMap samples [33]. CNV analysis using CNAT3 (Affymetrix) indicated that the *LEPR *gene locus contained potential CNV regions (CNVRs) in this Korean population (Additional file 1) [34]. In addition, we selected a copy number invariant region (CN2-2) in chromosome 2 (2q36.1) that exhibited the least copy number variation of the CN2-2 locus among the 90 individuals tested (Additional file 4, 5).

A potential CNV-affected region near rs2025804, upstream of the long isoform of LEPR (LEPRb, NM_002303), was chosen for experimental detection of DNA copy numbers using the QMPSF method [35]. The QMPSF reaction included the Factor VIII gene and CN2-2 as references [34,36,37]. The relative copy numbers of a target locus were calculated in relation to the copy numbers of the Factor VIII gene or CN2-2. These two normalizing genes yielded almost same results when used for calculating relative copy numbers. All samples were subjected to three rounds of QMPSF reactions. All reactions were performed in duplicate.

For the justification of further CNV calling, DNA copy numbers of Factor VIII gene were compared between male and female subjects (Additional file 4). Based on the relative copy numbers of Factor VIII gene to CN2-2 region, individuals of lower or higher copy number groups were assigned to be 1X or 2X of DNA copy number for Factor VIII gene. The copy numbers (1X or 2X) of Factor VIII gene was perfectly matched to individuals' sex.

Fluorescent primers were designed to target a specific region of the *LEPR *gene locus, encompassing over 216 kb of chromosome 1p31, as well as the Factor VIII gene and CN2-2 loci used for normalization of the QMPSF reactions. Following optimization of the PCR reaction, multiplex PCR was performed with the appropriate primers (Additional file 6). The amplicon size for the *LEPR *locus was 242bp or 245bp depending on the presence of a trinucleotide deletion (-/AGG, rs60086513, at chromosomal position 65603354-65603358) and 199bp for the Factor VIII gene. The DNA copy number of the *LEPR *locus in relative to the Factor VIII reference was calculated according to the peak height measurements of target and reference loci. The histogram of the *LEPR *copy numbers indicates two subpopulations among the non-diabetes subjects who had either low or high copy numbers at the *LEPR *locus (Additional file 2). Thus, median values of the DNA copy number relative to Factor VIII in non-diabetic male and female groups were used as cutoff values to call DNA copy number values in male and female groups, respectively. Finally, either a 'low (1X)' or 'high (2X)' DNA copy number value at the *LEPR *locus was assigned to all individuals.

### Quantitative real-time PCR

Total RNA was isolated from EBV-transformed lymphoblastoid cell lines (LCLs) that were generated from peripheral blood mononuclear cells of participants. cDNA was synthesized as previously described [38] and then used in SYBR green PCR with the appropriate primers (Additional file 6). The difference in threshold cycles (Ct) between *GAPDH *and the test genes was determined from three experiments, each performed in triplicate. The expression levels of the target genes were determined relative to the expression level of *GAPDH*.

### Statistical analyses

All data are presented as mean±SD. Student's *t *test was used to compare means of the baseline measurements between two groups. For statistical analysis, DNA copy numbers were dichotomized as '1' for low copy number and '2' for high copy number at the *LEPR *locus. Multiple regressions were performed for association analyses of metabolic phenotypes in non-diabetic subjects while adjusting for age and sex, which was then corrected by the Bonferroni method for multiple testing. Logistic regression analysis was used for calculating P-values, controlling for age, sex, and BMI as covariates. All analyses were performed with the SPSS 12.0 Statistical Software package.

## Abbreviations

CNV: copy number variation; QMPSF: quantitative multiplex PCR of short fluorescent fragment; LCL: lymphoblastoid cell line; LEPR: leptin receptor; T2DM: type 2 diabetes mellitus.

## Authors' contributions

JPJ developed the concept and wrote the paper. JPJ and BGH designed the study. SMS generated and analyzed experimental data, and drafted the manuscript. HYN and EJH participated in the QMPSF experiment. GMR extracted and analyzed CNV information from the chip data. HLK and BGH helped with interpretation of the results. BGH supervised the project. All authors read and approved the final manuscript.

## Supplementary Material

Additional file 1 **Copy number variations at the LEPR locus in a Korean population**. LEPR CNVs were analyzed using the Affymetrix 50 K SNP (A, B) and 5.0 SNP arrays (C) for 90 Korean individuals. (A) Affymetrix 50 K SNP array data was obtained from 90 individuals from the Korean HapMap project and then used to extract copy number information using the CNAT3 in reference to the Korean reference genome assembly of 90 individuals. For each SNP probe, standard deviation (STD) of the copy number (CN) values from 90 individuals was calculated and plotted along with the physical positions of SNP probes near the *LEPR *locus on chromosome 1. The STD of 0.25 was chosen as a cutoff value for CNV calling. A dashed box indicates copy number variations of the *LEPR *gene. (B) The Affymetrix 50 K SNP array data were analyzed to define CNV regions in reference to the Affymetrix reference genome assembly (provided by Affymetrix Inc.). The *P *value of < 0.01 was chosen to call CNVs. The CNV regions at the *LEPR *locus for 90 individuals were schematically depicted near the *LEPR *locus. A dashed box indicates copy number variations at the *LEPR *gene. Red bars: copy number gains, blue bars: copy number losses. (C) The whole genomes of 90 individuals were analyzed for CNV detection using the Affymetrix SNP array 5.0, and then compared with one specific reference genome (NA07357). The DNA-Chip Analyzer (dChip) was used for CNV detection http://www.dchip.org. dChip is a Windows software package for probe-level and high-level analysis of gene expression microarrays and SNP microarrays. dChip performed invariant set normalization on the 91 chip data (90 Koreans and one reference). Next it calculated model-based expression values. Copy number variations were inferred by applying median smoothing method. Only CNV regions covering *LEPR *gene were shown. Assuming that CN states are 2N in the reference genome, CN states indicate the copy numbers of corresponding genomic locus for each individual. Click here for file

Additional file 2 **Histogram of the E2 DNA content among non-T2DM subjects**. E2 DNA content was calculated relative to the DNA content at the Factor VIII gene locus from QMPSF data and then plotted to display the distribution pattern of E2 DNA copy numbers among the non-diabetes subjects. The median copy numbers in the men's and women's groups were used to divide them into two subpopulations of lower and higher E2 DNA copy number individuals. The E2 DNA content was then dichotomized into 'lower' or 'higher' copy number groups. Click here for file

Additional file 3 **Characteristics of case-control subjects**. *: Student's t-test for difference between low and high CN groups within either cases or controls. **: Student's t-test for difference between T2DM cases and non-T2DM controls. Click here for file

Additional file 4 **Justification of the QMPSF-based CNV calling using Factor VIII and CN2-2 references**. A) Experimental validation of the CN2-2 copy number invariant region using QMPSF. CNV analysis of the Affymetrix 50 k SNP array data for 90 individuals identified copy number invariant regions with the least standard deviation of copy number (CN) values in the CNAT3 analysis. Relative copy numbers of the CN2-2 region were calculated in relative to the Factor VIII gene reference from 35 individuals. Error bars indicate standard deviations from three independent QMPSF reactions. Each reaction was performed in duplicate. Relative copy numbers of CN2-2 region to Factor VIII gene exhibited 7.3% of coefficient of variation (CV), suggesting that CN2-2 can be used as a good internal control of further QMPSF reactions. B) Copy number distribution of Factor VIII gene in male and female subjects. Total individuals (n = 1,202) were clearly divided into two subgroups (male and female groups) by relative DNA copy numbers of Factor VIII gene to CN2-2 region. Individuals of lower or higher copy number groups were assigned to be 1X or 2X for Factor VIII gene. The DNA copy numbers (1X or 2X) of Factor VIII gene were perfectly matched to individuals' sex. Click here for file

Additional file 5 **Copy number invariant regions**. a: Copy number values (CN) of each SNP probe were obtained from CNV analysis of 90 individuals using the CNAT3. b: Average of copy number values of 90 individuals for each SNP probe. c: Standard deviation of copy number values of 90 individuals for each SNP probe. d: Average copy number values of all SNP probes for 90 individuals within the CNV region. e: Standard deviations of all probes for 90 individuals within the CNV region. Click here for file

Additional file 6 **Primers used in QMPSF and real-time PCR**. Click here for file
