# Supplementary material for: Copy number variation at leptin receptor gene locus associated with metabolic traits and the risk of type 2 diabetes mellitus
Source: BMC Genomics. 2010 Jul 12;11:426. doi: 10.1186/1471-2164-11-426 (PMC2996954; doi:10.1186/1471-2164-11-426)
Supplement: Additional file 1 — Copy number variations at the LEPR locus in a Korean population. LEPR CNVs were analyzed using the Affymetrix 50 K SNP (A, B) and 5.0 SNP arrays (C) for 90 Korean individuals. (A) Affymetrix 50 K SNP array data was obtained from 90 individuals from the Korean HapMap project and then used to extract copy number information using the CNAT3 in reference to the Korean reference genome assembly of 90 individuals. For each SNP probe, standard deviation (STD) of the copy number (CN) values from 90 individuals was calculated and plotted along with the physical positions of SNP probes near the LEPR locus on chromosome 1. The STD of 0.25 was chosen as a cutoff value for CNV calling. A dashed box indicates copy number variations of the LEPR gene. (B) The Affymetrix 50 K SNP array data were analyzed to define CNV regions in reference to the Affymetrix reference genome assembly (provided by Affymetrix Inc.). The P value of < 0.01 was chosen to call CNVs. The CNV regions at the LEPR locus for 90 individuals were schematically depicted near the LEPR locus. A dashed box indicates copy number variations at the LEPR gene. Red bars: copy number gains, blue bars: copy number losses. (C) The whole genomes of 90 individuals were analyzed for CNV detection using the Affymetrix SNP array 5.0, and then compared with one specific reference genome (NA07357). The DNA-Chip Analyzer (dChip) was used for CNV detection http://www.dchip.org. dChip is a Windows software package for probe-level and high-level analysis of gene expression microarrays and SNP microarrays. dChip performed invariant set normalization on the 91 chip data (90 Koreans and one reference). Next it calculated model-based expression values. Copy number variations were inferred by applying median smoothing method. Only CNV regions covering LEPR gene were shown. Assuming that CN states are 2N in the reference genome, CN states indicate the copy numbers of corresponding genomic locus for each individual. [file 1471-2164-11-426-S1.PPT]

## Slide 1
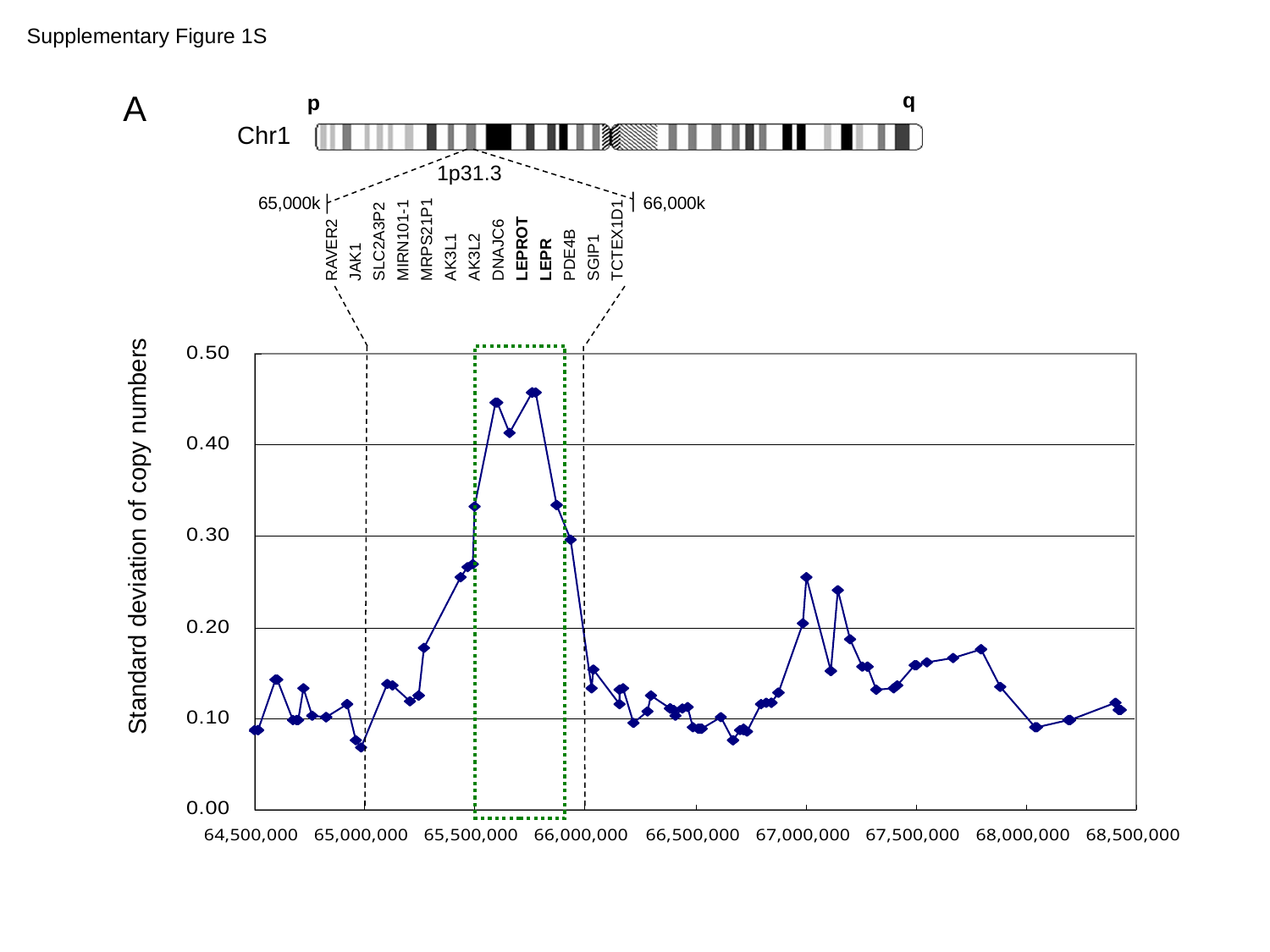

Supplementary Figure 1S
A
RAVER2
JAK1
SLC2A3P2
MIRN101-1
MRPS21P1
AK3L1
AK3L2
DNAJC6
LEPROT
LEPR
PDE4B
SGIP1
TCTEX1D1
q
p
Chr1
1p31.3
65,000k
66,000k
Standard deviation of copy numbers

## Slide 2
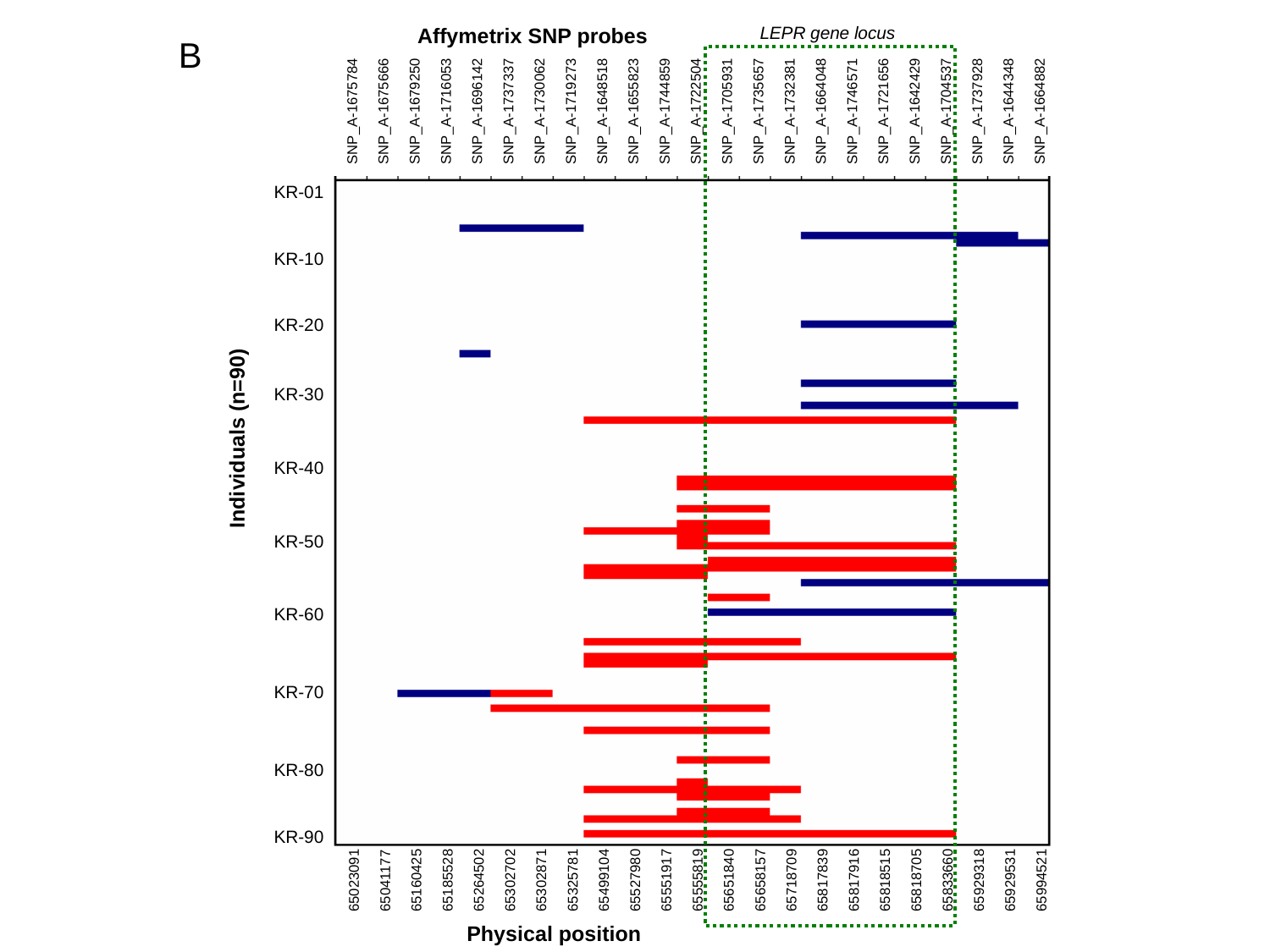

SNP_A-1675784
SNP_A-1675666
SNP_A-1679250
SNP_A-1716053
SNP_A-1696142
SNP_A-1737337
SNP_A-1730062
SNP_A-1719273
SNP_A-1648518
SNP_A-1655823
SNP_A-1744859
SNP_A-1722504
SNP_A-1705931
SNP_A-1735657
SNP_A-1732381
SNP_A-1664048
SNP_A-1746571
SNP_A-1721656
SNP_A-1642429
SNP_A-1704537
SNP_A-1737928
SNP_A-1644348
SNP_A-1664882
LEPR gene locus
Affymetrix SNP probes
B
KR-01
KR-10
KR-20
KR-30
KR-40
KR-50
KR-60
KR-70
KR-80
KR-90
Individuals (n=90)
65023091
65041177
65160425
65185528
65264502
65302702
65302871
65325781
65499104
65527980
65551917
65555819
65651840
65658157
65718709
65817839
65817916
65818515
65818705
65833660
65929318
65929531
65994521
Physical position

## Slide 3
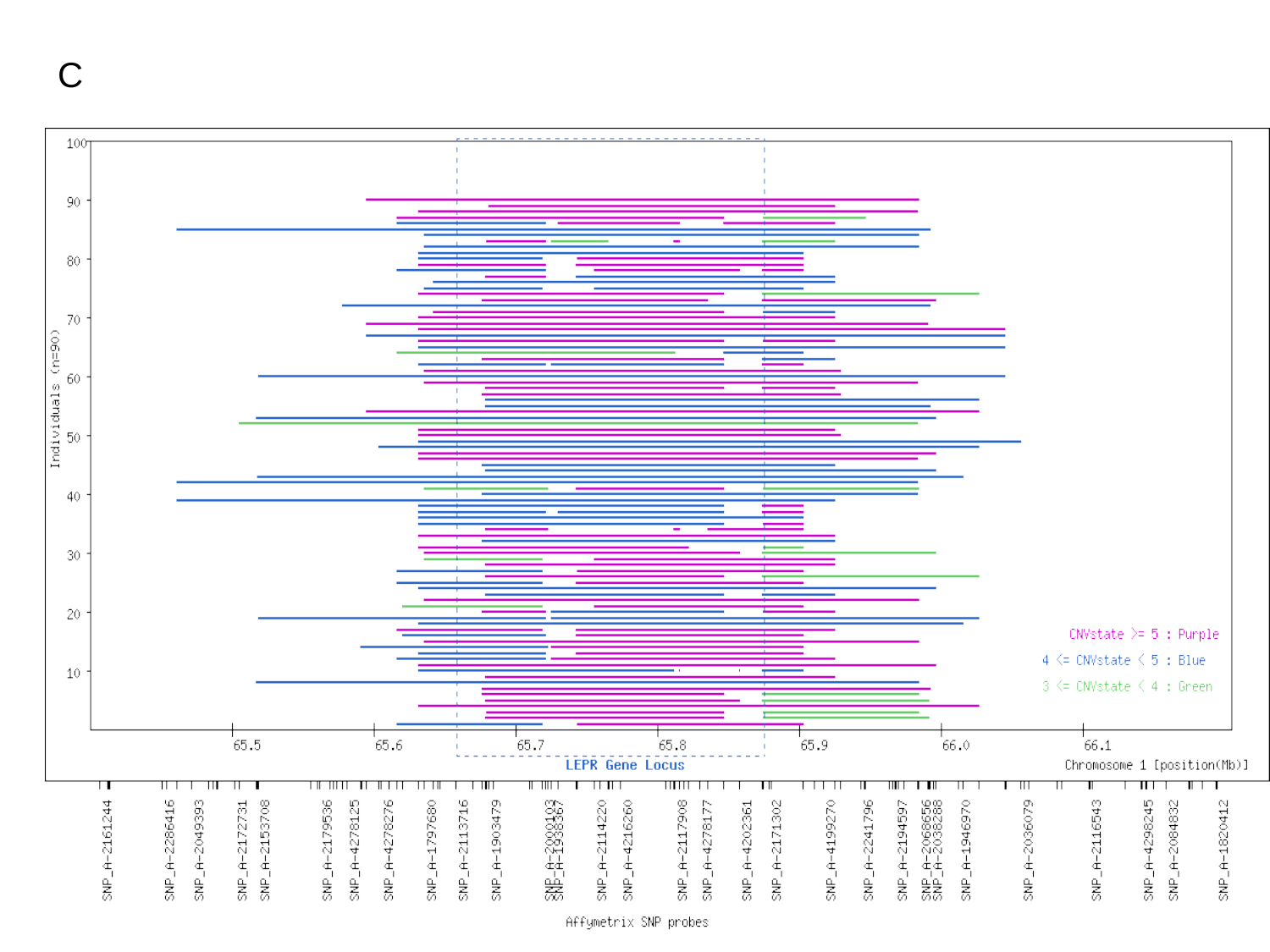

C
