# Supplementary material for: Copy number variation at leptin receptor gene locus associated with metabolic traits and the risk of type 2 diabetes mellitus
Source: BMC Genomics. 2010 Jul 12;11:426. doi: 10.1186/1471-2164-11-426 (PMC2996954; doi:10.1186/1471-2164-11-426)
Supplement: Additional file 2 — Histogram of the E2 DNA content among non-T2DM subjects. E2 DNA content was calculated relative to the DNA content at the Factor VIII gene locus from QMPSF data and then plotted to display the distribution pattern of E2 DNA copy numbers among the non-diabetes subjects. The median copy numbers in the men's and women's groups were used to divide them into two subpopulations of lower and higher E2 DNA copy number individuals. The E2 DNA content was then dichotomized into 'lower' or 'higher' copy number groups. [file 1471-2164-11-426-S2.PPT]

## Slide 1
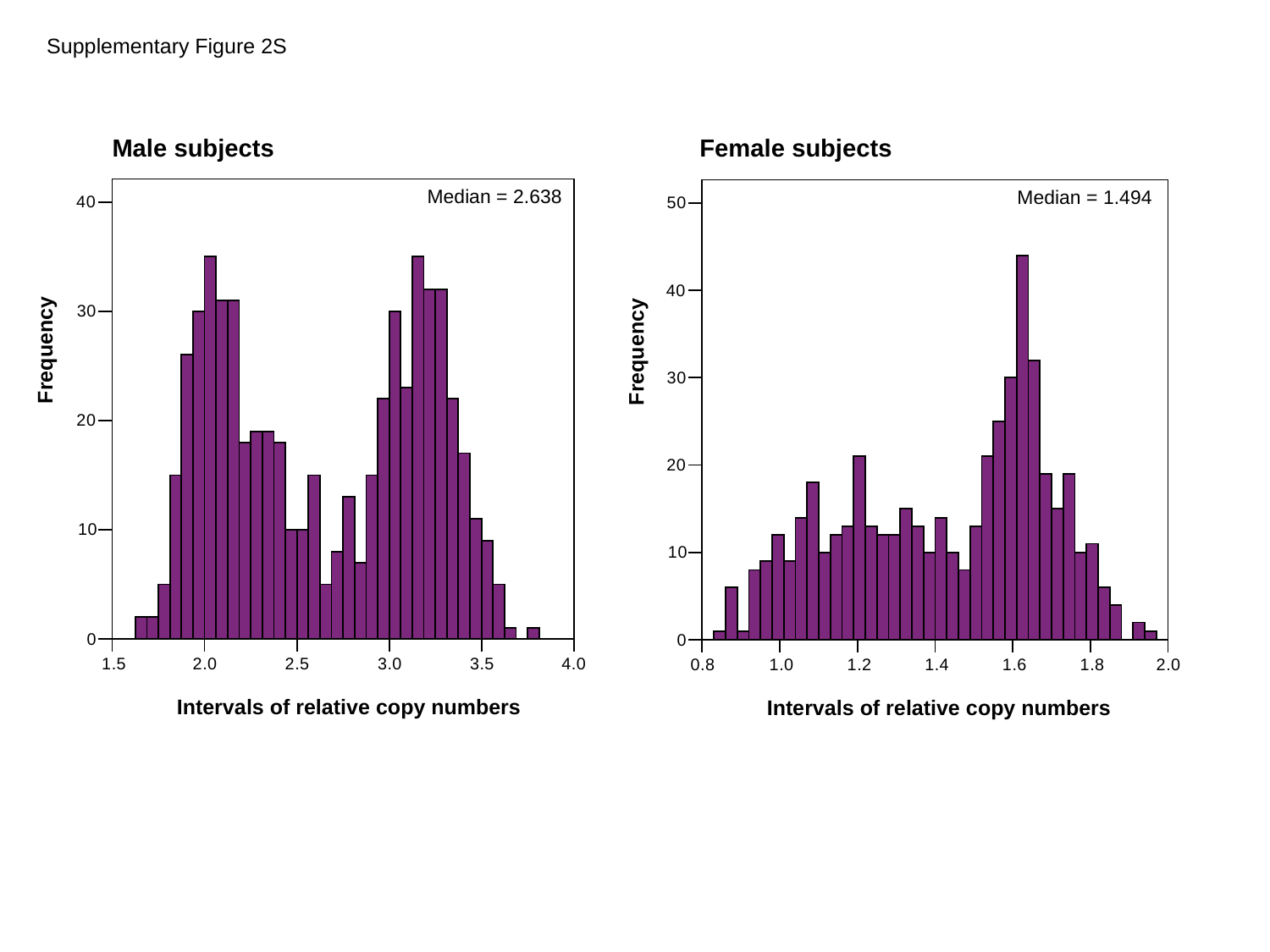

Supplementary Figure 2S
Male subjects
Median = 2.638
Frequency
Intervals of relative copy numbers
Female subjects
Median = 1.494
Frequency
Intervals of relative copy numbers
