# Supplementary material for: Copy number variation at leptin receptor gene locus associated with metabolic traits and the risk of type 2 diabetes mellitus
Source: BMC Genomics. 2010 Jul 12;11:426. doi: 10.1186/1471-2164-11-426 (PMC2996954; doi:10.1186/1471-2164-11-426)
Supplement: Additional file 4 — Justification of the QMPSF-based CNV calling using Factor VIII and CN2-2 references. A) Experimental validation of the CN2-2 copy number invariant region using QMPSF. CNV analysis of the Affymetrix 50 k SNP array data for 90 individuals identified copy number invariant regions with the least standard deviation of copy number (CN) values in the CNAT3 analysis. Relative copy numbers of the CN2-2 region were calculated in relative to the Factor VIII gene reference from 35 individuals. Error bars indicate standard deviations from three independent QMPSF reactions. Each reaction was performed in duplicate. Relative copy numbers of CN2-2 region to Factor VIII gene exhibited 7.3% of coefficient of variation (CV), suggesting that CN2-2 can be used as a good internal control of further QMPSF reactions. B) Copy number distribution of Factor VIII gene in male and female subjects. Total individuals (n = 1,202) were clearly divided into two subgroups (male and female groups) by relative DNA copy numbers of Factor VIII gene to CN2-2 region. Individuals of lower or higher copy number groups were assigned to be 1X or 2X for Factor VIII gene. The DNA copy numbers (1X or 2X) of Factor VIII gene were perfectly matched to individuals' sex. [file 1471-2164-11-426-S4.PPT]

## Slide 1
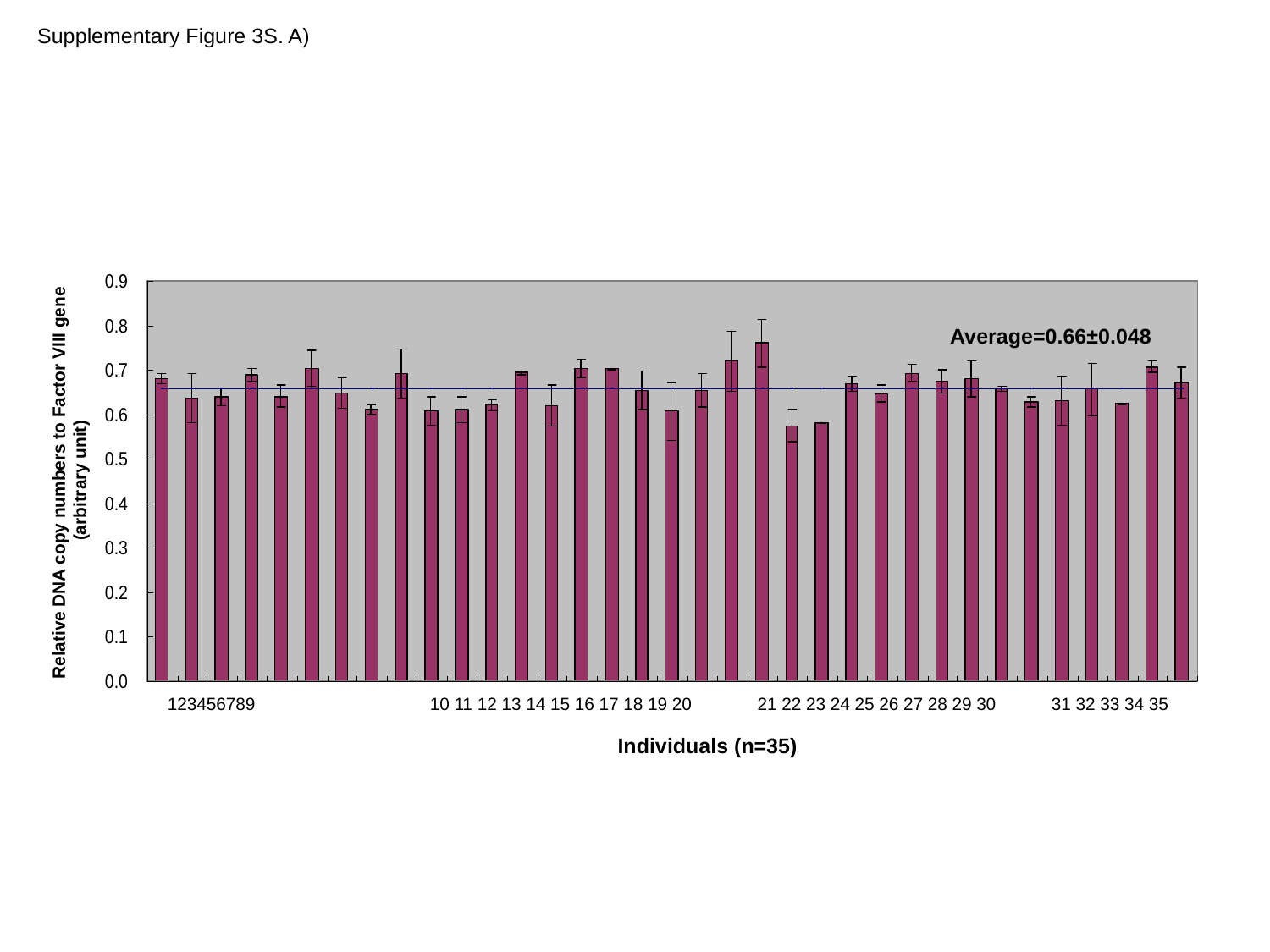

Supplementary Figure 3S. A)
Relative DNA copy numbers to Factor VIII gene
(arbitrary unit)
Average=0.66±0.048
123456789
10 11 12 13 14 15 16 17 18 19 20
21 22 23 24 25 26 27 28 29 30
31 32 33 34 35
Individuals (n=35)

## Slide 2
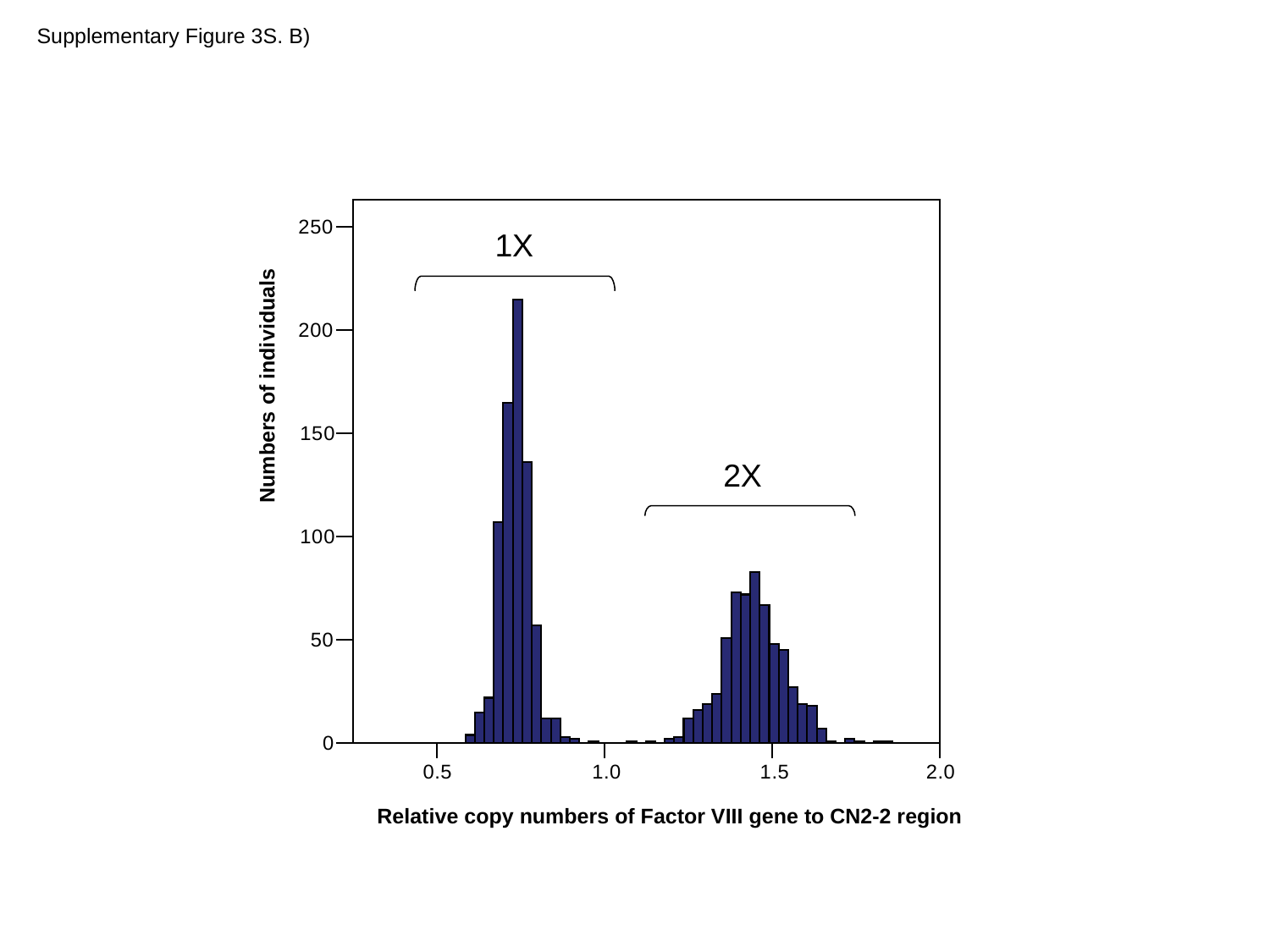

Supplementary Figure 3S. B)
1X
Numbers of individuals
2X
Relative copy numbers of Factor VIII gene to CN2-2 region
